# Health service providers experience of psycho-emotional violence and associated factors among urban hospitals in Eastern Ethiopia

**DOI:** 10.3389/fpubh.2024.1361243

**Published:** 2024-05-03

**Authors:** Abebe Tolera, Adisu Birhanu Weldesenbet, Lemma Demissie Regassa, Biruk Shalmeno Tusa, Bedasa Taye Merga, Mandaras Tariku, Abera Cheru, Daniel Birhanie Enyew, Assefa Tola Gemeda

**Affiliations:** ^1^Department of Epidemiology and Biostatistics, School of Public Health, College of Health and Medical Sciences, Haramaya University, Harar, Ethiopia; ^2^Department of Psychiatry, College of Health and Medical Sciences, Haramaya University, Harar, Ethiopia; ^3^School of Environmental Health Science, College of Health and Medical Sciences, Haramaya University, Harar, Ethiopia

**Keywords:** psycho-emotional violence, psycho-emotional health, workplace violence coping mechanisms, health professionals, eastern Ethiopia

## Abstract

**Background:**

Psycho-emotional violence, a type of workplace violence targeting healthcare workers, varies across countries, occasions, and professions in the healthcare sector. Unfortunately, there is a scarcity of comprehensive studies focusing on violence against healthcare workers in Ethiopia, which may also encompass psycho-gender-based emotional violence against healthcare workers. Therefore, there is a compelling need for in-depth research to address this gap and develop effective strategies to mitigate psycho-emotional violence in the healthcare sector in Ethiopia, especially in the eastern region. Hence, we aimed to identify the prevalence of and factors associated with workplace psycho-emotional violence against healthcare providers in eastern Ethiopia.

**Methods:**

This institution-based cross-sectional study was conducted among 744 health professionals working in urban public hospitals in eastern Ethiopia. Multistage stratified random sampling was used, and data were collected using a standardized structured tool adopted from the WHO workplace violence assessment tool. Binary and multivariable logistic regression analyses were employed to identify factors associated with psycho-emotional workplace violence. Adjusted odds ratio (OR) with 95% confidence interval (CI) was reported, and a *p*-value of 0.05 was used as the cut-off point to declare significance.

**Results:**

Workplace psycho-emotional violence was reported by 57.39% of the healthcare workers. The absence of guidelines for gender-based abuse [AOR = 35.62, 95% CI:17.47, 72.64], presence of measures that improve surroundings (class lighting and privacy) [AOR = 0.58, 95% CI: 0.35, 0.98], training on workplace violence coping mechanism [AOR = 0.16, 95%CI: 0.26, 0.98], spending more than 50% of their time with HIV/AIDS patients [AOR = 1.96, 95%CI:1.05, 3.72], and spending more than 50% of their time with psychiatric patients [AOR = 1.92, 95%CI:1.08, 3.43] were factors significantly associated with workplace violence against health professionals.

**Conclusion:**

The prevalence of workplace psycho-emotional violence against health professionals in eastern Ethiopia was relatively high. Improving the working environment decreases the chance of workplace violence; however, there is a lack of guidelines for gender-based violence, the absence of training on coping mechanisms, and spending more time with psychiatric and HIV/AIDS patients’ increases workplace violence. We recommend that health institutions develop gender abuse mitigation guidelines and provide training on coping mechanisms.

## Introduction

Mental health, including emotional, psychological, and social well-being, is important at every stage of our life (1, 2). It enables people to realize their full potential, work productively and make meaningful contributions to their communities (2–4). Furthermore, proper psycho-emotional health involves a normal emotional, behavioral, and social maturity to a person (5). Work place violence derails such mental health of healthcare workers. Work place violence against healthcare workers is a problem of growing interest in research and has been poorly prioritized in healthcare systems (6). Violence against healthcare workers are varied and a complex phenomenon (7).

Psycho-emotional abuse (Verbal abuse, sexual and racial harassment, bullying/mobbing and other threats) was the most reported worked place violence in many countries (7, 8). Psycho-emotional abuse is any non-physical pattern of behavior that intentionally harms an individual’s mental state and undermines their ability to reach their full potential (5, 9). It results from a person’s dislike towards a person or object, especially when they feel like that person or object did something wrong (10–12). Psycho-emotional abuse may be overt or covert and can be tricky to spot – even by the person experiencing it (11). The abuser’s behaviors are deliberate and intentional and often rooted in envy, fragility, and aggression. It may manifest in forms of harassment, intimidation, or other threatening disruptive behavior that may occurs at the work site (5, 9).

Verbal abuse leaves no visible scars, but the emotional damage can be devastating (6). Today, doctors pay more attention to a person’s emotions and behaviors as indicators of potential health issues than ever before. Acts of violence and other injuries is currently the third-leading cause of fatal occupational injuries in the United States. Psycho-emotional violence at work has many negative impacts both at the organizational and individual level, such as the erosion of individual’s self-esteem, debases their sense of achievement, diminishes their sense of belonging, prevents their healthy and vigorous development, and takes away the individual’s well-being (11, 13). Some of the specific consequences include: decreased job performance and job satisfaction, confusion, self-blame, depression, inability to concentrate, lack of motivation, procrastination, low self-esteem, fear of failure, hopelessness, worthlessness, and self-sabotage (11).

The prevalence of psycho-emotional violence varies between countries and across different professions. In countries like Egypt (7) and Jordan (14) verbal abuse was reported to be 69.5% among nurses and (63.5%) among physicians, respectively. In Barbados, psycho-emotional abuse (60% verbal abuse, 19% bullying, 7% sexual harassment and 3% racial harassment) were found to be high among medical staffs (8). The prevalence of verbal abuse and sexual harassment was found to be a little bit higher in Riyadh (79.5 and 76.5% respectively) (15) and Saudi Arabia (75.6%) (16). Similarly, verbal abuse was found to be high among nurses working in psychiatry hospital in Jordan (71.9%) (17).

Being female (8); age, being nurse and night work (18); working when understaffed, working directly with volatile people, drug and alcohol abuse, inadequate security, long waits for service (19); working in psychiatry hospitals or psychiatry department (17) were identified as major factors for such violence.

Cases of severe psycho-emotional trauma, if left unmanaged, may lead to retaliatory violence, chronic illness, or even suicide and fear of future events. Although there have been numerous studies on psycho-emotional, there is no consensus regarding the current status of psychological violence directed at health care workers in in low income counties like Ethiopia. This study aimed to identify service providers’ experience of psycho-emotional violence and its associated factors in eastern Ethiopia.

## Methods

### Study design, setting and period

A cross-sectional study was conducted among health professionals working at public hospitals in eastern Ethiopia, including Harar and Dire Dawa, located 525 km and 515 km from the capital of Ethiopia (Addis Ababa), respectively. Harari region has one general and one comprehensive and specialized university hospital, whereas the Dire Dawa city administration has one general and one referral hospital that serves millions of people located in the eastern part of Ethiopia. According to the Harari National Region Health Bureau report/evidence, approximately 560 healthcare workers were working in these health institutions. Similarly, the two public hospitals in Dawa had approximately 400 healthcare providers at the time of the study. This study was conducted from January 18 to February 10, 2022.

### Study population and sampling procedure

Multi-stage sampling was used to randomly select health professionals working in selected public hospitals in eastern Ethiopia based on their institutional identification number. All health professionals who had been in these public hospitals for more than 6 months were included in this study. All healthcare professionals on annual leave, or maternity leave, and those who were critically ill during data collection were excluded from the study. The sample size is calculated using the single population proportion formula by taking the prevalence of psycho-emotional trauma among health professionals from previous studies (prevalence in % 95 confidence level and 5% margin of error using a study conducted in Gonder Referral Hospital) (20). The final sample size was proportionally allocated to each health facility based on the actual number of healthcare workers. The health professionals were then stratified by professional category as doctors/health officers, nurses/midwives (nurses, midwives, nursing assistants, and theatre attendants), laboratory technicians, and pharmacists. The sample size in each hospital was proportionally allocated to each healthcare professional category according to their number. Finally, simple random sampling was used to select the required sample.

### Data collection and quality control procedures

Data were collected by health professionals (BSc Public Health officers) using a structured questionnaire prepared by the World Health Organization (21) which contains both open- and closed-ended questions as well as other workplace violence assessment tools from other similar studies (7, 22–24) that were adopted and modified in such a way that could meet the objectives of this study. The questionnaire contained questions on psycho-emotional violence, individual factors, work-related factors, and organizational factors that directly met the objectives of the study. Data quality was ensured through the training of data collectors, pre-testing of questionnaires, electronic data collection (Kobo collect), and supervision of the data collection process. In addition, respondents were encouraged to respond to all items in the questionnaire within the time they devoted to minimizing a large non-response rate. The completeness and consistency of the data were checked by the data collectors immediately after receiving the questionnaire from each participant. The questionnaire was pretested at Haramaya Hospital in 5% of the sample.

### Variables and measurements

The main outcome variable was psycho-emotional violence (verbal abuse, bullying/mobbing, harassment, and threats) experienced by study participants in the last 12 months, answered with yes or no questions.

#### Workplace violence

Incidents when staff members are abused, threatened, or assaulted in circumstances related to their work, including commuting to and from work, involving an explicit or implicit challenge to their safety, well-being, or health.

#### Psychological violence (emotional abuse)

The intentional use of power, including the threat of physical force, against another person or group that can harm physical, mental, spiritual, moral, or social development. These include verbal abuse, bullying/mobbing, harassment, and threats. This was measured with a yes or no answer if the health professional experienced one of these violence.

#### Abuse

Behavior that humiliates degrades or otherwise indicates a lack of respect for the dignity and worth of an individual. This was measured with a yes or no answer if the health professional experienced one of the violence.

#### Bullying/Mobbing

Repeated and overtime offensive behavior through vindictive, cruel, or malicious attempts to humiliate or undermine an individual or group of employees. This was measured with a yes or no answer if the health professional experienced one of this violence.

#### Harassment

Any conduct based on age, disability, HIV status, domestic circumstances, sex, sexual orientation, gender reassignment, race, color, language, religion, politics, trade union or other opinion or belief, national or social origin, association with a minority, property, birth, or another status that is unreciprocated or unwanted, which affects the dignity of men and women at work.

#### Sexual harassment

Any unwanted, unreciprocated, and unwelcome behavior of a sexual nature that is offensive to the person involved, and causes that person to be threatened, humiliated, or embarrassed. This was measured with a yes or no answer if the health professional experienced one of this violence.

#### Racial harassment

Any threatening conduct based on race, color, language, national origin, association with a minority, birth, or another status that is unreciprocated or unwanted, which affects the dignity of women and men at work. This was measured with a yes or no answer if the health professional experienced one of these violence.

#### Threat

Promised use of physical force or power (i.e., psychological force) results in fear of physical, sexual, psychological harm, or other negative consequences to the targeted individuals or groups. This was measured with a yes or no answer if the health professional experienced one of these violence.

#### Emotional health

Refers to the emotional quality experienced by an individual. The emotional health outcomes of WPV include emotional exhaustion, detachment, sadness, frustration, and anger.

### Data processing and analysis

Data from the Kobo collection were downloaded into Excel and exported to Stata 16. Data were cleaned and analyzed using descriptive summary measures, such as percentage, frequencies, and mean/median and standard deviation (SD), as appropriate, and presented using tables and graphs. Logistic analysis with a 95% confidence interval was used to determine the factors associated with psycho-emotional violence and its determinants. Variables with *p*-values less than or equal to 0.25 were entered into a multivariable logistic regression model. Hosmer-Lemeshow’s goodness-of-fit tests were used to determine the goodness of fit for the final model. In multivariate analysis, variables with a *p*-value less than 0.05 were considered to be significantly associated with psycho-emotional violence.

## Results

### Health workers characteristics

A total of 744 healthcare providers were interviewed. More than half of the study participants 53.76 and 58.84% were males and married, respectively. The median age of the study participants was 31(+7) years, and the median work experience was 6 (+7.30SD) years. A total of 17 (2.28%) were senior managers (Table 1).

**Table 1:** Socio-demographic characteristics of the study participants, eastern Ethiopia, 2022.

Variables	Category	Frequency	Percent
Sex	Male	400	53.76
	Female	344	46.24
Marital status	Married	438	58.87
	Single	289	38.84
	Separate	15	2.02
	Widowed	2	0.27
Profession	Nurse	345	46.37
	Physician	113	15.19
	Pharmacist	74	9.95
	Midwifery	64	8.60
	Medical laboratory	64	8.60
	Radiologist	19	2.55
	Psychiatrist	17	2.28
	Others*	48	6.45
Position	Staff	674	90.59
	Line manger	50	6.72
	Senior manger	17	2.28
	Others	3	0.40
Ward	Laboratory Unit	68	9.14
	Pediatric ward	47	6.33
	Surgical ward	40	5.38
	Medical ward	37	4.97
	Emergency ward	99	13.31
	ICU	60	8.06
	MCH	17	2.28
	OPD	66	8.87
	Obstetrics/gynaecology ward	75	10.08
	Operating room	39	5.24
	Pharmacy unit	74	9.95
	Radiology unit	22	2.96
	Others**	100	13.44

Others*: anaesthesia, environmental health, health officers, Optometrists, counsellors. Others**: Chronic follow-up, Dental OPD, Dermatology Unit, Psychiatry unit, and management.

### Prevalence of psycho-emotional violence

Psycho-emotional violence was reported by 57.39% of the HCWs. Verbal violence was reported by 71.37% (531/744) of participants. In contrast, bullying/mobbing was reported by 13.71% (102/744) of participants. Sexual and racial harassment was reported by 0.94% (7/744) and 9% (65/744) of health professionals, respectively.

### Sources of psycho-emotional violence against health professionals

According to the findings of this study, patient families accounted for 61.6% (434) of verbal violence cases, while patients themselves accounted for 40% (282) of verbal violence cases. Regarding the main source of bullying/mobbing, patients’ relatives accounted for 5.8% (41) of the main sources and patients themselves accounted for 3.1% (22). Similarly, the main sources of racial harassment were relatives of relatives, 38 (5.4%), followed by staff members (3.5%, 25). Furthermore, the main source of sexual harassment was staff members (0.42%) (3), followed by patients themselves (0.29%) (2) (Table 2).

**Table 2:** Sources of psycho-emotional violence against healthcare providers, eastern Ethiopia, 2022.

Sources		Verbal abuse	Bullying/mobbing	Racial harassment	Sexual harassment
Patient/client	No	248	80	43	5
	Yes	282	22	22	2
Relatives of patient/client	No	96	61	27	7
	Yes	434	41	38	0
Staff member	No	463	68	40	4
	Yes	67	34	25	3
Management/ supervisor	No	509	97	55	7
	Yes	21	5	10	0
External colleague/worker	No	525	102	63	7
	Yes	5	0	2	0
General public	No	524	102	57	5
	Yes	6	0	8	2

### Actions taken after psycho-emotional violence incident

The majority of study participants (57.5%, 404) said they did not take any action following psycho-emotional violence against them, of which 69.3% (280) took no action following verbal abuse against them. Similarly, about 25.5% (179) of the study participants told the person to stop psycho-emotional violence, of which 83.2% (149) said to person following verbal abuse against them (Additional file 1).

### Knowledge of workplace violence policies in their health facilities

**Figure 1:**
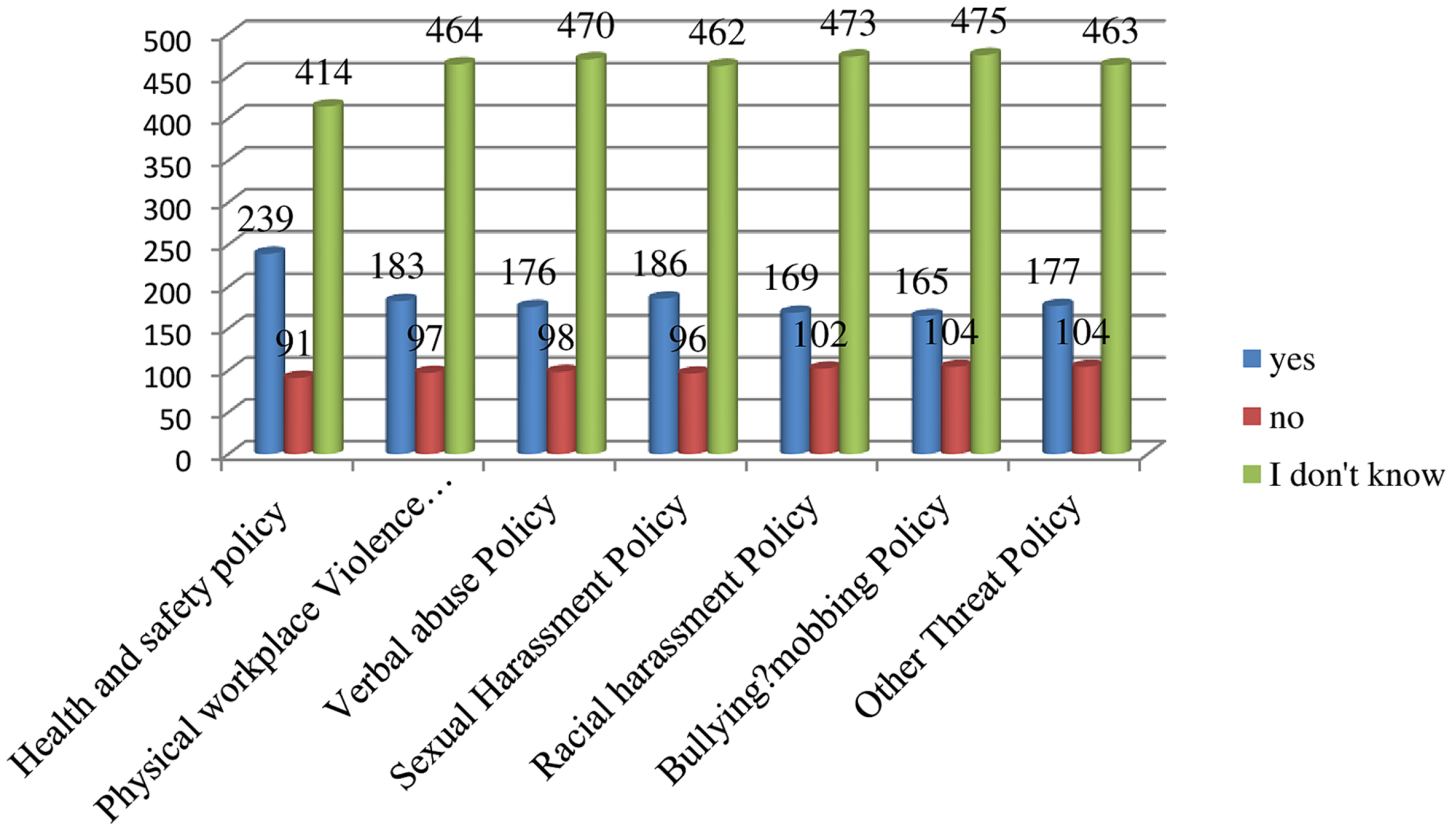
Knowledge of study participants whether their employer developed workplace violence policies in their health facilities, eastern Ethiopia, 2022.

The majority of study participants said they did not know whether their employer had developed specific policies on health safety (55.65%), physical workplace violence (62.37%), verbal abuse (63.17%), bullying/mobbing (63.84%), sexual harassment (62.10%), racial harassment (63.58%), and other threats (62.23%) (Figure 1).

### Available measures to deal with psycho-emotional workplace violence

**Figure 2:**
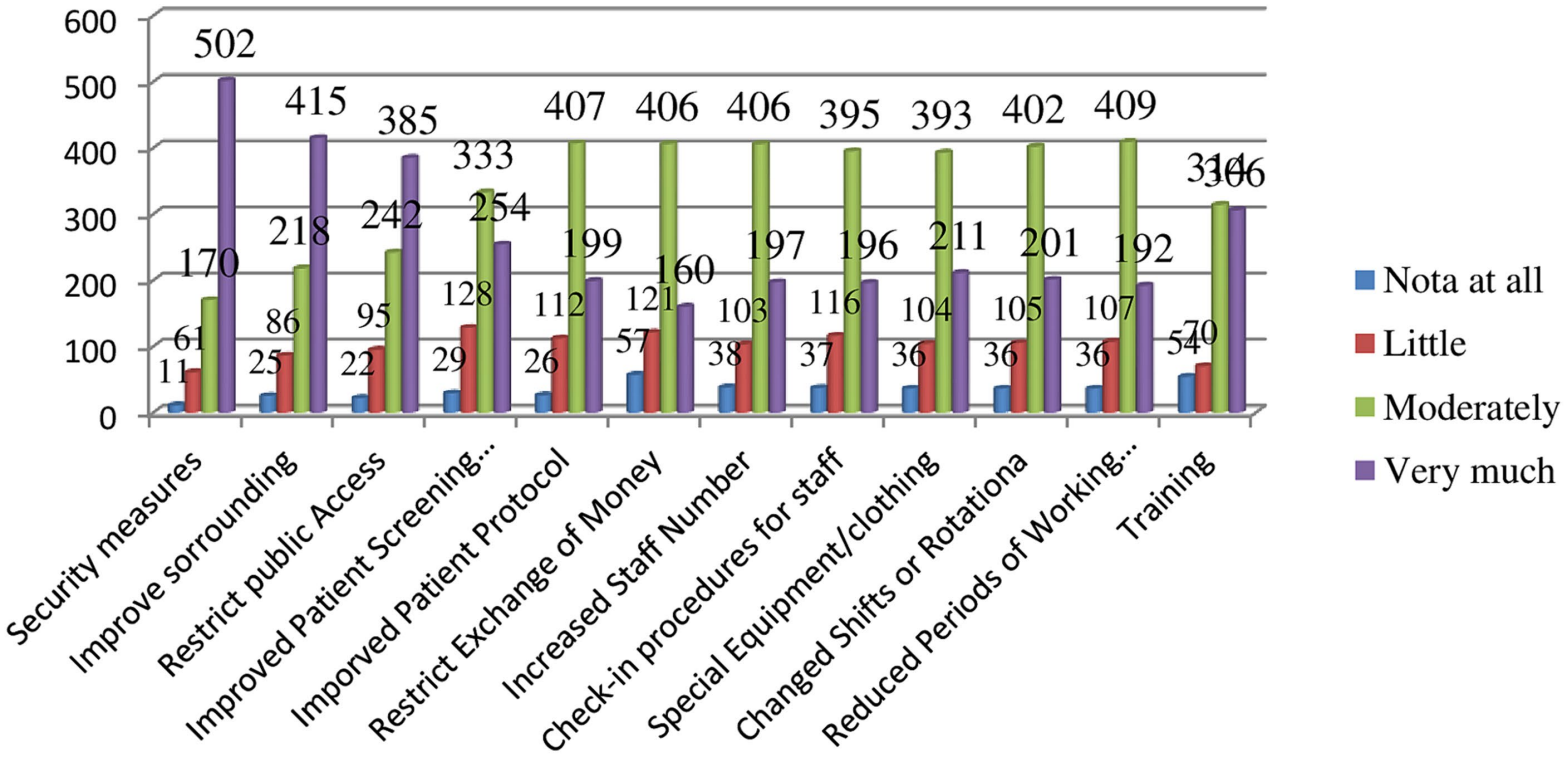
Available measures to deal with psycho-emotional workplace violence, eastern Ethiopia, 2022.

The majority of the study participants (64.47%; 502/744) said that there was a security structure to deal with such psycho-emotional workplace violence (Figure 2).

### Factors associated with psycho-emotional violence

Multivariable logistic regression analysis showed that the absence of guidelines for gender-based abuse [AOR = 35.62, 95% CI (17.47, 72.64)], presence of measures that improve surroundings (class lighting and privacy) [AOR = 0.58, 95% CI (0.35, 0.98)], training on WPV coping mechanism [AOR = 0.16, 95%CI (0.26, 0.98)], spending more than 50% of their time with HIV/AIDS patients (AOR = 1.96, 95%CI [1.05, 3.72]), and spending more than 50% of their time with psychiatric patients [AOR = 1.92, 95%CI (1.08, 3.43)] were factors significantly associated with WPV against health professionals.

Accordingly, the odds of WPV were 35.62 times higher among health professionals in settings that had guidelines for gender-based abuse as compared to those in settings that had no guidelines [AOR = 35.62, 95% CI (17.47, 72.64)].

The odds of experiencing WPV decreased by 42% for health professionals in settings where measures that improve surroundings (class lighting and privacy) were taken as compared to in settings where such measures were not implemented [AOR = 0.58, 95% CI (0.35, 0.98)].

The odds of experiencing WPV decreased by 84% for health professionals who took training on violence coping mechanisms compared to those who did not receive training, keeping other variables in the model constant (AOR = 0.16, 95%CI [0.26, 0.98]).

The odds of WPV increased by 98% among health professionals spending most of their working time with HIV/AIDS patients compared to their counterparts while adjusting for other variables in the model (AOR = 1.98, 95%CI [1.05, 3.72]).

The odds of WPV were increased by 92% among health professionals who spend more than 50% of their time with psychiatric patients as compared to their counterparts holding other variables in the model constant [AOR = 1.92, 95%CI (1.08, 3.43)] (Table 3).

**Table 3:** Factors associated with psycho-emotional workplace violence against health professionals working in Public Health Facilities of eastern Ethiopia, 2022.

Variable	Category	WPV		COR (95% CI)	*p*-value	AOR (95% CI)	*p*-value
		Yes	No				
Marital status	Married	245	193	Ref.	Ref.	Ref.	Ref.
	Never married	167	122	1.08 (0.79, 1.46)	0.622	0.84 (0.49, 1.45)	0.55
	Separated/divorced/widowed	15	2	5.91 (1.33, 26.15)	0.019	4.71 (0.75, 29.69)	0.09
Moved from other places to current address	Yes	252	229	1.83 (1.34, 2.50)	<0.001	0.68 (0.38, 1.23)	0.203
	No	175	87	Ref.	Ref.	Ref.	Ref.
Work experience	Mean(±SD)	8.07 (7.65)	7.42 (6.79)	1.01 (0.99, 1.03)	0.231	1.01 (0.97,1.05)	0.612
Presence of a procedure for reporting violence	Yes	269	256	Ref.	Ref.	Ref.	Ref.
	No	158	60	2.51 (1.78, 3.53)	<0.001	1.31 (0.74, 2.34)	0.356
Presence of guidelines for gender based abuse	Yes	252	306	Ref.	Ref.	Ref.	Ref.
	No	175	11	19.32 (10.27, 36.33)	<0.001	35.62 (17.47, 72.64)	<0.001
Security measures to deal with violence (guards)	Yes	25	30	0.59 (0.34, 1.03)	0.065	0.42 (0.17, 1.06)	0.67
	No	402	287	Ref.	Ref.	Ref.	Ref.
Improve surroundings to deal with violence (lighting)	Yes	293	192	0.70 (0.52, 0.95)	0.023	0.58 (0.35, 0.98)	0.043
	No	134	125	Ref.	Ref.	Ref.	Ref.
Training on WPV coping mechanism	Yes	411	313	0.33 (0.11, 0.99)	0.048	0.16 (0.26, 0.98)	0.048
	No	16	4	Ref.	Ref.	Ref.	Ref.
Spent more than 50% of their working time with terminally ill patients	Yes	145	135	1.44 (1.07, 1.95)	0.016	1.18 (0.68, 2.07)	0.545
	No	282	182	Ref.	Ref.	Ref.	Ref.
Spent more than 50% of their time with HIV/AIDS patients	Yes	203	209	2.14 (1.58, 2.88)	<0.001	1.98 (1.05, 3.72)	0.034
	No	224	108	Ref.	Ref.	Ref.	Ref.
Spent more than 50% of their time with psychiatric patients	No	271	242	Ref.	Ref.	Ref.	Ref.
	Yes	156	75	1.86 (1.34, 2.57)	<0.001	1.92 (1.08, 3.43)	0.027
Changes structure and organization of the facility	No	298	250	Ref.	Ref.	Ref.	Ref.
	Yes	129	67	1.62 (1.15, 2.27)	0.006	1.45 (0.86, 2.43)	0.163
Decrease in the number of staff	No	403	286	Ref.	Ref.	Ref.	Ref.
	Yes	24	31	1.82 (1.05, 3.17)	0.034	2.17 (0.89, 5.29)	0.090
Action was taken on incidents of abuse	No	193	301	2.12 (1.04, 4.32)	0.039	1.64 (0.68, 3.91)	0.266
	Yes	19	14	Ref.	Ref.	Ref.	Ref.

## Discussion

This study assessed the magnitude of WPV and its associated factors among health professionals in eastern Ethiopia. Accordingly, the finding showed 57.39% (95% CI: 53.75–60.98%) experienced WPV. This finding is in line with the prevalence reported in Brazil (25), Saudi Arabia (16), Egypt, South Africa, Thailand, and Portugal (26) and 5 European countries (Poland, the Czech Republic, the Slovak Republic, Turkey, and Spain) (23) but higher than findings from studies in southern Ethiopia (27, 28). The higher prevalence of WPV in the current study might be due to the differences in the study population. A previous study from Southern Ethiopia assessed violence against nurses, whereas the current study assessed violence against all categories of health professionals. Evidently, stress and violence are widespread in health sector where doctors, nurses and social workers experience repeated violence (25).

Most health professionals had experienced verbal abuse (71%). This is in agreement with previous studies (23–25), and might be because it is easily committed in the form of shouting, belittling, and offensive or obscene language, harassing remarks.

Similar to previous studies (29, 30), patients and patient relatives were the main sources of violence. This could be explained by the fact that patients and patient relatives might have frequent contact with health workers, which may increase the risk of health workers experiencing violence. This study also identified horizontal violence from fellow coworkers. This can be attributed to line of authority and nightshift work as stated by female healthcare workers. Female healthcare workers reported they were sexually abused by their coworkers during night shift.

This study also identified factors that were significantly associated with WPV among health workers. Improving surroundings (lighting, noise, and privacy) decreases workplace violence against health workers. This could be explained by the fact that those measures help to stop behavior that may develop into future violence and limit public access to restricted areas, which in turn reduces unnecessary contact between physicians and patients as well as patient relatives. In addition, such measures reduce the entrance of perpetrators into potential working rooms without the permission of health workers.

The absence of guidelines for gender-based abuse makes WPV against healthcare professionals 35 times more likely. This might be because the policy will inform perpetrators and victims about the kind of behavior (violence, intimidation, bullying, and harassment) considered inappropriate and unacceptable in the workplace and what to do when incidents occur. Therefore, if such guidelines are not implemented, health workers become confused about reporting incidents and the procedure that will be followed when an incident is reported, which raises the probability that violence is repeated.

The odds of experiencing WPV were lowered by 84% among health professionals who received training on violence coping mechanisms compared to those who did not receive training. This finding is in agreement with that of a previous study (31), which stated that training is a key component of reducing workplace aggression. This is because training increases the confidence and ability of health workers to recognize signs of aggression and practice evidence-based interventions to de-escalate agitated patients. In addition, training familiarizes health professionals with their legal responsibilities, risk assessment, effective communication, conflict management, and self-defence.

Working in the psychiatry department was significantly associated with WPV. The odds of experiencing WPV were higher among health professionals who spent more than 50% of their time with psychiatric patients (32) with psychiatric patients. This finding is supported by previous studies (33, 34), and this could be due to the verbal and physical aggression shown by most psychiatric patients. In addition, psychiatric patients are addicted to alcohol and illicit drugs, which might increase their aggressive behavior that makes them commit violence against health workers. This can contributes to deterioration of health care workers’ work life and health, impacts patient care delivery and hinders recruitment and retention of healthcare workers (35).

Caring for HIV/AIDS patients was another variable that was significantly associated with WPV among health professionals. Health workers who spend much of their working time with patients with HIV/AIDS are more likely to develop WPV. This finding is supported by evidence from previous studies (25, 36) where healthcare providers engaged in chronic patient care experience unprecedented psycho-emotional violence such as harassment and verbal abuse. This might be explained by comorbidities and complex medical conditions that lead to the deterioration of physical and psychological health among HIV/AIDS patients. These situations put healthcare professionals caring for HIV/AIDS patients under stressful work conditions.

The main drawback of the current study is the possibility of recall bias, as it requires participants to remember their previous experience with WPV.

## Conclusion

The prevalence of Psycho-emotional violence among health professionals in eastern Ethiopia was relatively high. Measures that improve surroundings (class lighting and privacy) decrease the magnitude of WPV, whereas the absence of guidelines for gender-based abuse, lack of training on WPV coping mechanisms, and spending more than 50% of their time with psychiatric and HIV/AIDS patients increases with WPV against health professionals. We recommend that health institutions develop gender abuse mitigation guidelines and provide special training on the coping mechanisms of WPV. In addition, special protection measures are needed for workers in the psychiatry and HIV/AIDS departments.

## Data availability statement

The raw data supporting the conclusions of this article will be made available by the authors, without undue reservation.

## Ethics statement

The studies involving humans were approved by Ethical clearance was obtained from the institutional Health Research Ethics Review Committee (IHRERC/262/2020) of the College of Health and Medical Sciences, Haramaya University. The studies were conducted in accordance with the local legislation and institutional requirements. Written informed consent for participation in this study was provided by the participants' legal guardians/next of kin.

## Author contributions

AT: Conceptualization, Data curation, Formal analysis, Funding acquisition, Methodology, Resources, Writing – original draft, Writing – review & editing. AW: Conceptualization, Data curation, Formal analysis, Funding acquisition, Methodology, Software, Writing – original draft, Writing – review & editing. LR: Conceptualization, Data curation, Formal analysis, Funding acquisition, Methodology, Resources, Software, Validation, Writing – original draft, Writing – review & editing. BT: Conceptualization, Formal analysis, Funding acquisition, Methodology, Writing – original draft, Writing – review & editing. BM: Conceptualization, Data curation, Formal analysis, Methodology, Writing – original draft, Writing – review & editing. MT: Conceptualization, Data curation, Formal analysis, Methodology, Writing – original draft, Writing – review & editing. AC: Conceptualization, Data curation, Formal analysis, Methodology, Writing – original draft, Writing – review & editing. DE: Conceptualization, Data curation, Formal analysis, Funding acquisition, Methodology, Writing – original draft, Writing – review & editing. AG: Conceptualization, Data curation, Formal analysis, Funding acquisition, Investigation, Methodology, Visualization, Writing – original draft, Writing – review & editing.

## References

[1] Prince M Patel V Saxena S Maj M Maselko J Phillips MR. No health without mental health. Lancet. (2007) 370:859–77. doi: 10.1016/S0140-6736(07)61238-0 17804063

[2] Goetzel RZ Roemer EC Holingue C Fallin MD McCleary K Eaton W. Mental health in the workplace: a call to action proceedings from the mental health in the workplace: public health summit. J Occup Environ Med. (2018) 60:322–30. doi: 10.1097/JOM.0000000000001271, PMID: 29280775 PMC5891372

[3] Keyes CL Lopez SJ. Toward a science of mental health In: Oxford handbook of positive psychology, Eds. C.R. Snyder and Shane J. Lopez. (New York: Oxford university press) (2009). 89:89–95.

[4] Brundtland GH. Mental health in the 21st century. Bull World Health Organ. (2000) 78:503–505. PMID: 10885158 PMC2560741

[5] Rothstein MA. The occupational safety and health act at 50: introduction to the special section. Am J Public Health. (2020) 110:613–4. doi: 10.2105/AJPH.2020.305623, PMID: 32267740 PMC7144434

[6] National Observatory of Aggressions to Physicians (ONAM) Workgroup Garrote-Díaz JM Becerra-Becerra A Bendaña-Jácome J Casero-Cuevas L Garrote-Cuevas G. National report on aggressions to physicians in Spain 2010–2015: violence in the workplace-ecological study. BMC Res Notes. (2018) 11:347. doi: 10.1186/s13104-018-3393-7, 29866171 PMC5985576

[7] Abdellah RF Salama KM. Prevalence and risk factors of workplace violence against health care workers in emergency department in Ismailia, Egypt. Pan Afr Med J. (2017) 26:21. doi: 10.11604/pamj.2017.26.21.10837 28451000 PMC5398248

[8] Abed M Morris E Sobers-Grannum N. Workplace violence against medical staff in healthcare facilities in Barbados. Occup Med (Oxford, England). (2016) 66:580–3. doi: 10.1093/occmed/kqw073, PMID: 27371658

[9] Kumari A Kaur T Ranjan P Chopra S Sarkar S Baitha U. Workplace violence against doctors: characteristics, risk factors, and mitigation strategies. J Postgrad Med. (2020) 66:149–54. doi: 10.4103/jpgm.JPGM_96_20, PMID: 32675451 PMC7542052

[10] Strecker PJ. I wish that he hit me: The experiences of people who have been Psychoemotionally abused and have Psychoemotionally abused others Melbourne, Australia: Victoria University Research Repository (VURR), (2012).

[11] Iwaniec D. The emotionally abused and neglected child: Identification, assessment and intervention: a practice handbook. England: John Wiley & Sons (2006).

[12] Paat Y-F Markham C Peskin M. Psycho-emotional violence, its association, co-occurrence, and Bidirectionality with cyber, physical and sexual violence. J Child Adolesc Trauma. (2020) 13:365–80. doi: 10.1007/s40653-019-00283-z, PMID: 33269037 PMC7683755

[13] Loring MT. Emotional abuse, © 2024 American Psychological Association. 750 first street NE. Washington, DC 20002–4242: Lexington Books/Macmillan (1994).

[14] Alhamad R Suleiman A Bsisu I Santarisi A Al Owaidat A Sabri A. Violence against physicians in Jordan: an analytical cross-sectional study. PLoS One. (2021) 16:e0245192. doi: 10.1371/journal.pone.0245192, PMID: 33493170 PMC7833172

[15] Alharbi FF Alzneidi NA Aljbli GH Morad SA Alsubaie EG Mahmoud MA. Workplace violence among healthcare Workers in a Tertiary Medical City in Riyadh: a cross-sectional study. Cureus. (2021) 13:e14836. doi: 10.7759/cureus.14836, PMID: 34123608 PMC8191849

[16] Alshahrani M Alfaisal R Alshahrani K Alotaibi L Alghoraibi H Alghamdi E. Incidence and prevalence of violence toward health care workers in emergency departments: a multicenter cross-sectional survey. Int J Emerg Med. (2021) 14:71. doi: 10.1186/s12245-021-00394-1, PMID: 34906080 PMC8903599

[17] Al-Omari H Abu Khait A Al-Modallal H Al-Awabdeh E Hamaideh S. Workplace violence against nurses working in psychiatric hospitals in Jordan. Arch Psychiatr Nurs. (2019) 33:58–62. doi: 10.1016/j.apnu.2019.08.002, PMID: 31711595

[18] Alsaleem SA Alsabaani A Alamri RS Hadi RA Alkhayri MH Badawi KK. Violence towards healthcare workers: a study conducted in Abha City, Saudi Arabia. J Fam Community Med. (2018) 25:188–93. doi: 10.4103/jfcm.JFCM_170_17 PMC6130164 30220849

[19] Alkorashy HA Al Moalad FB. Workplace violence against nursing staff in a Saudi university hospital. Int Nurs Rev. (2016) 63:226–32. doi: 10.1111/inr.12242, PMID: 26830364

[20] Yenealem DG Woldegebriel MK Olana AT Mekonnen TH. Violence at work: determinants & prevalence among health care workers, Northwest Ethiopia: an institutional based cross sectional study. Ann Occup Environ Med. (2019) 31:8. doi: 10.1186/s40557-019-0288-6, PMID: 30992993 PMC6449966

[21] WHO. (2003) Workplace Violence in the Health Sector, Country Case Studies. Rsearch Instruments, Survey Questionnaire, English; Joint Programme on Workplace Violence in the Health Sector.WHO TEAM, Social Determinants of Health (SDH). Editor: ILO/ICN/WHO/PSI Joint Programme on Workplace Violence in the Health Sector. GENEVA 2003. Number of pages 14. Available at: https://www.who.int/publications/m/item/workplace-violence-in-the-health-sector---country-case-study-research-instruments---survey-questionnaire (Accessed on January 4, 2024).

[22] Pinar T Acikel C Pinar G Karabulut E Saygun M Bariskin E. Workplace violence in the health sector in Turkey: a national study. J Interpers Violence. (2017) 32:2345–65. doi: 10.1177/0886260515591976, PMID: 26124224

[23] Babiarczyk B Turbiarz A Tomagová M Zeleníková R Önler E Sancho CD. Reporting of workplace violence towards nurses in 5 European countries–a cross-sectional study. Int J Occup Med Environ Health. (2020) 33:325–38. doi: 10.13075/ijomeh.1896.01475, PMID: 32235948

[24] d'Ettorre G Pellicani V. Workplace violence toward mental healthcare workers employed in psychiatric wards. Saf Health Work. (2017) 8:337–42. doi: 10.1016/j.shaw.2017.01.004, PMID: 29276631 PMC5715456

[25] Santos JD Meira KC Coelho JC Dantas ESO Oliveira LVE Oliveira JSA. Work-related violences and associated variables in oncology nursing professionals. Ciênc Saúde Colet. (2021) 26:5955–66. doi: 10.1590/1413-812320212612.14942021, PMID: 34909988

[26] Di Martino V. Workplace violence in the health sector. Country case studies Brazil, Bulgaria, Lebanon, Portugal, South Africa, Thailand and an additional Australian study. Ginebra: Organización Internacional del Trabajo. (2002):3–42.

[27] Weldehawaryat HN Weldehawariat FG Negash FG. Prevalence of workplace violence and associated factors against nurses working in public health facilities in southern Ethiopia. Risk Manag Healthc Policy. (2020) 13:1869–77. doi: 10.2147/RMHP.S264178, PMID: 33061720 PMC7538000

[28] Fute M Mengesha ZB Wakgari N Tessema GA. High prevalence of workplace violence among nurses working at public health facilities in southern Ethiopia. BMC Nurs. (2015) 14:1–5. doi: 10.1186/s12912-015-0062-1 25767412 PMC4357058

[29] Likassa T Gudissa T Mariam C. Assessment of factors associated with workplace violence against nurses among referral hospitals of Oromia regional state. Ethiop J Health Med Nurs. (2017) 35:22–31.

[30] Fehler-Cabral G Campbell R Patterson D. Adult sexual assault survivors’ experiences with sexual assault nurse examiners (SANEs). J Interpers Violence. (2011) 26:3618–39. doi: 10.1177/0886260511403761, PMID: 21602203

[31] Anderson L FitzGerald M Luck L. An integrative literature review of interventions to reduce violence against emergency department nurses. J Clin Nurs. (2010) 19:2520–30. doi: 10.1111/j.1365-2702.2009.03144.x, PMID: 20553349

[32] Lehmann LS McCormick RA Kizer KW. A survey of assaultive behavior in veterans health administration facilities. Psychiatr Serv. (1999) 50:384–9. doi: 10.1176/ps.50.3.384, PMID: 10096644

[33] Ferri P Silvestri M Artoni C Di Lorenzo R. Workplace violence in different settings and among various health professionals in an Italian general hospital: a cross-sectional study. Psychol Res Behav Manag. (2016) 9:263–75. doi: 10.2147/PRBM.S114870, PMID: 27729818 PMC5042196

[34] Su-hsing SL Gerberich SG Waller LA Anderson A McGovern P. Work-related assault injuries among nurses. Epidemiology. (1999) 10:685–91. doi: 10.1097/00001648-199911000-00007 10535781

[35] Needham I Kingma M O’Brien-Pallas L McKenna K Tucker R Oud N, editors. Workplace violence in the health sector. The Netherlands: KAVANAH, (2008).

[36] Musengamana V Adejumo O Banamwana G Mukagendaneza MJ Twahirwa TS Munyaneza E. Workplace violence experience among nurses at a selected university teaching hospital in Rwanda. Pan Afr Med J. (2022) 41:64. doi: 10.11604/pamj.2022.41.64.30865, PMID: 35371373 PMC8933447

