# Supplementary material for: Health service providers experience of psycho-emotional violence and associated factors among urban hospitals in Eastern Ethiopia
Source: Front Public Health. 2024 May 3;12:1361243. doi: 10.3389/fpubh.2024.1361243 (PMC11100414; doi:10.3389/fpubh.2024.1361243)
Supplement: Supplementary file 1 [file Table_1.DOCX]

Additional file -1: Actions taken after psycho-emotional violence incident, eastern Ethiopia, 2022

| More than 50% of time spent working with any of the following type of specialities | | **Verbal abuse** | **Bullying/mobbing** | **Racial harassment** | **Sexual harassment** | |
| --- | --- | --- | --- | --- | --- | --- |
| Took no action | No | 249 | 31 | 4 | 15 |  |
|  | Yes | 280 | 71 | 3 | 50 |  |
| Tried to pretend it never happened | No | 492 | 96 | 7 | 59 |  |
|  | Yes | 37 | 6 | - | 6 |  |
| Told the person to stop | No | 380 | 78 | 7 | 59 |  |
|  | Yes | 149 | 24 | - | 6 |  |
| Told friends/family | No | 504 | 99 | 6 | 64 |  |
|  | Yes | 25 | 3 | 1 | 1 |  |
| Told a colleague | No | 488 | 101 | 7 | 61 |  |
|  | Yes | 41 | 1 | - | 4 |  |
| Reported it to a senior staff member | No | 461 | 95 | 4 | 60 |  |
|  | Yes | 68 | 7 | 3 | 5 |  |
| Sought counseling | No | 420 | 93 | 7 | 61 |  |
|  | Yes | 109 | 9 | - | 4 |  |
| Sought help from the union | No | 525 | 102 | 7 | 65 |  |
|  | Yes | 4 | - | - | - |  |
| Sought help from the association | No | 528 | 102 | 7 | 65 |  |
|  | Yes | 1 | - | - | - |  |
| Transferred to another position | No | 526 | 100 | 7 | 65 |  |
|  | Yes | 3 | 2 | - | - |  |
| Completed incident/accident form | No | 529 | 102 | 7 | 65 |  |
|  | Yes | - | - | - | - |  |
| Pursued prosecution | No | 526 | 102 | 7 | 65 |  |
|  | Yes | 3 | - | - | - |  |
| Completed a compensation claim | No | 528 | 102 | 7 | 65 |  |
|  | Yes | 1 | - | - | - |  |
| Others* | No | 525 | 102 | 7 | 65 |  |
|  | Yes | 4 | - | - | - |  |

Others*: defended myself, insult back
